# Supplementary material for: The Implication of Serum Autoantibodies in Prognosis of Canine Mammary Tumors
Source: Animals (Basel). 2022 Sep 18;12(18):2463. doi: 10.3390/ani12182463 (PMC9495273; doi:10.3390/ani12182463)
Supplement: Supplementary file 1 [file animals-12-02463-s001.zip › Figure S1.pdf]

Figure S1

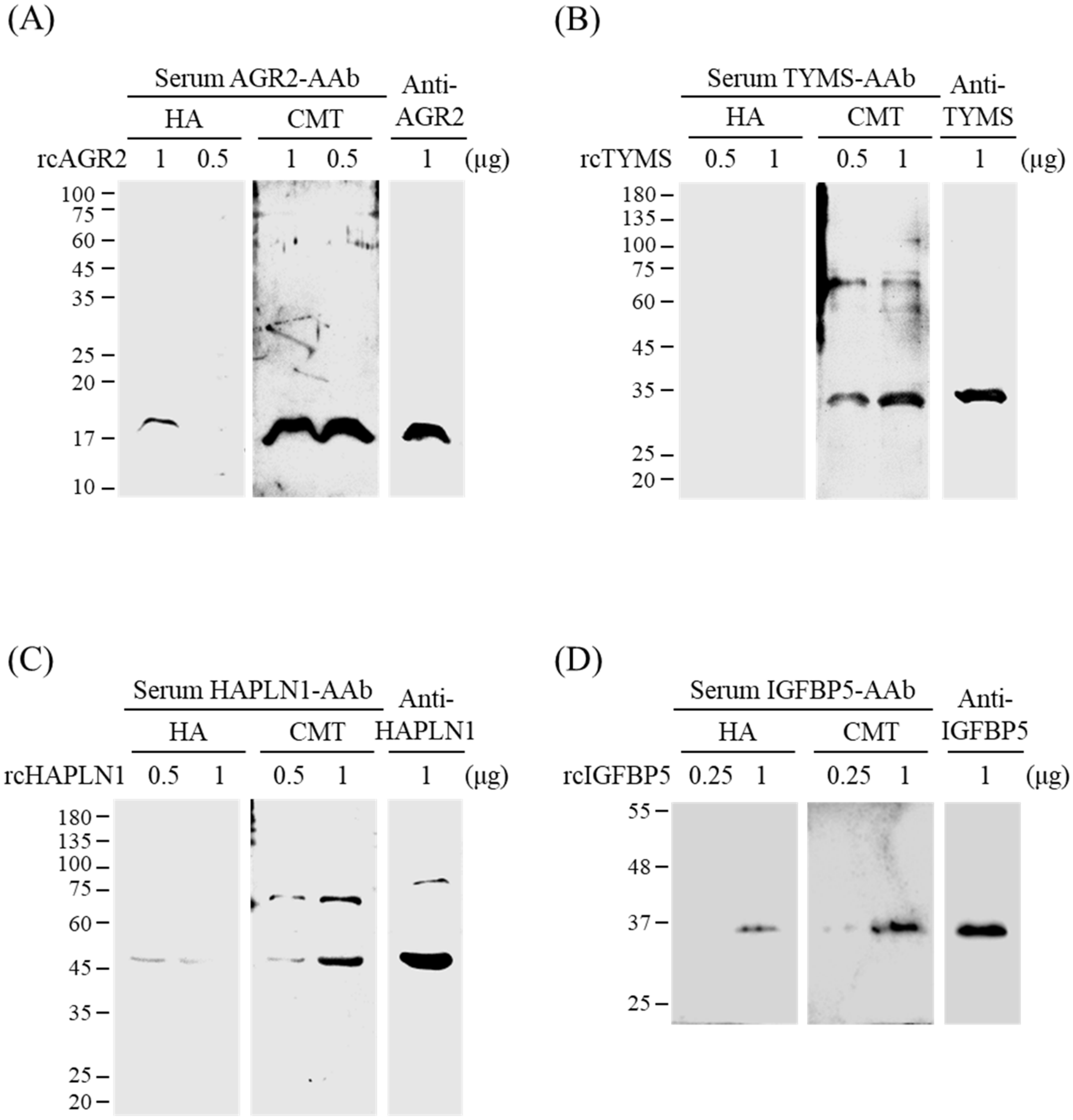

Figure S1. Detection of serum autoantibodies to TYMS, HAPLN1, or IGFBP5 by using immunoblotting. Recombinant proteins (A) AGR2, (B) TYMS, (C) HAPLN1, or (D) IGFBP5 resolved by SDS-PAGE and transferred onto PVDF membranes were blotted individually with 50-fold diluted sera collected from malignant CMT cases or healthy controls (denoted HA). Serum autoantibodies reactive to the recombinant proteins, denoted AGR2-AAb, TYMS-AAb, HAPLN1-AAb, and IGFBP5-AAb, were sequentially detected by a goat anti-canine IgG antibody conjugated with HRP. Immunoblotting of the recombinant proteins with a specific antibody was used as a positive control. All the images were acquired with the same condition setting.
