# Supplementary material for: The Implication of Serum Autoantibodies in Prognosis of Canine Mammary Tumors
Source: Animals (Basel). 2022 Sep 18;12(18):2463. doi: 10.3390/ani12182463 (PMC9495273; doi:10.3390/ani12182463)
Supplement: Supplementary file 1 [file animals-12-02463-s001.zip › Figure S2.pdf]

Figure S2

(A)

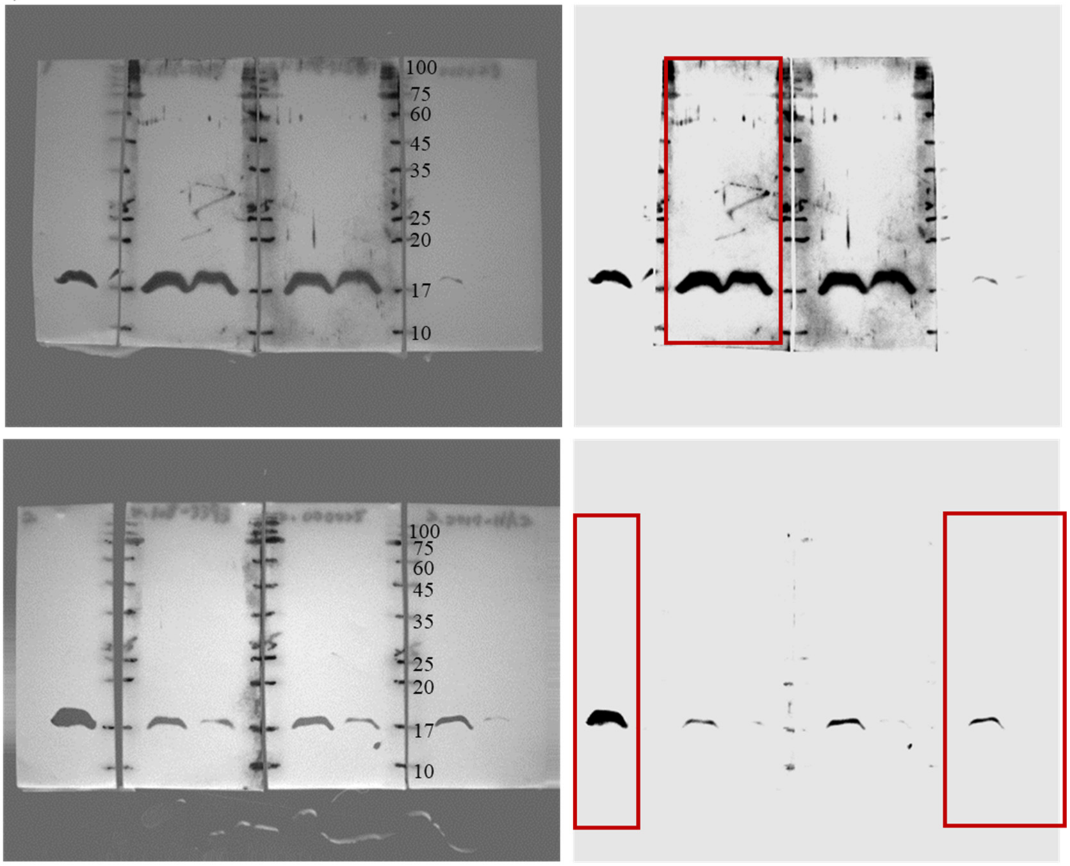

(B)

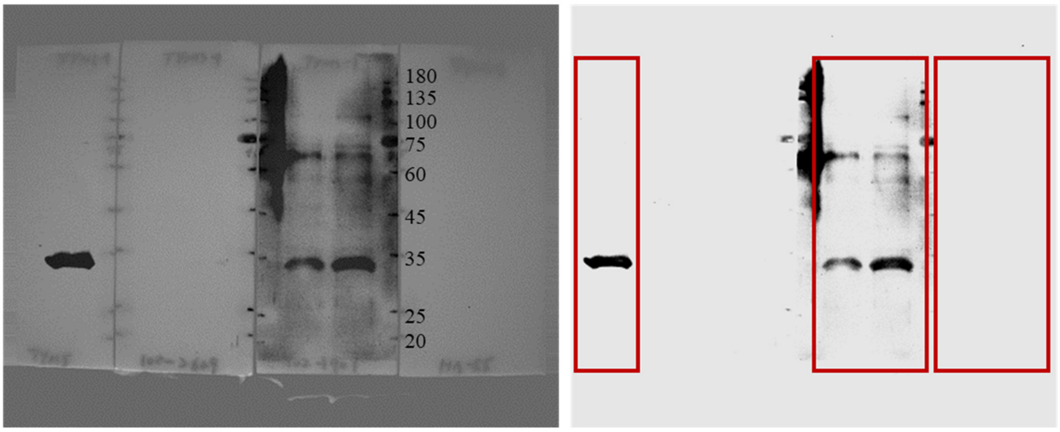

Figure S2. (A) Original images of Figure S1 (A). (B) Original images of Figure S1 (B).

(C)

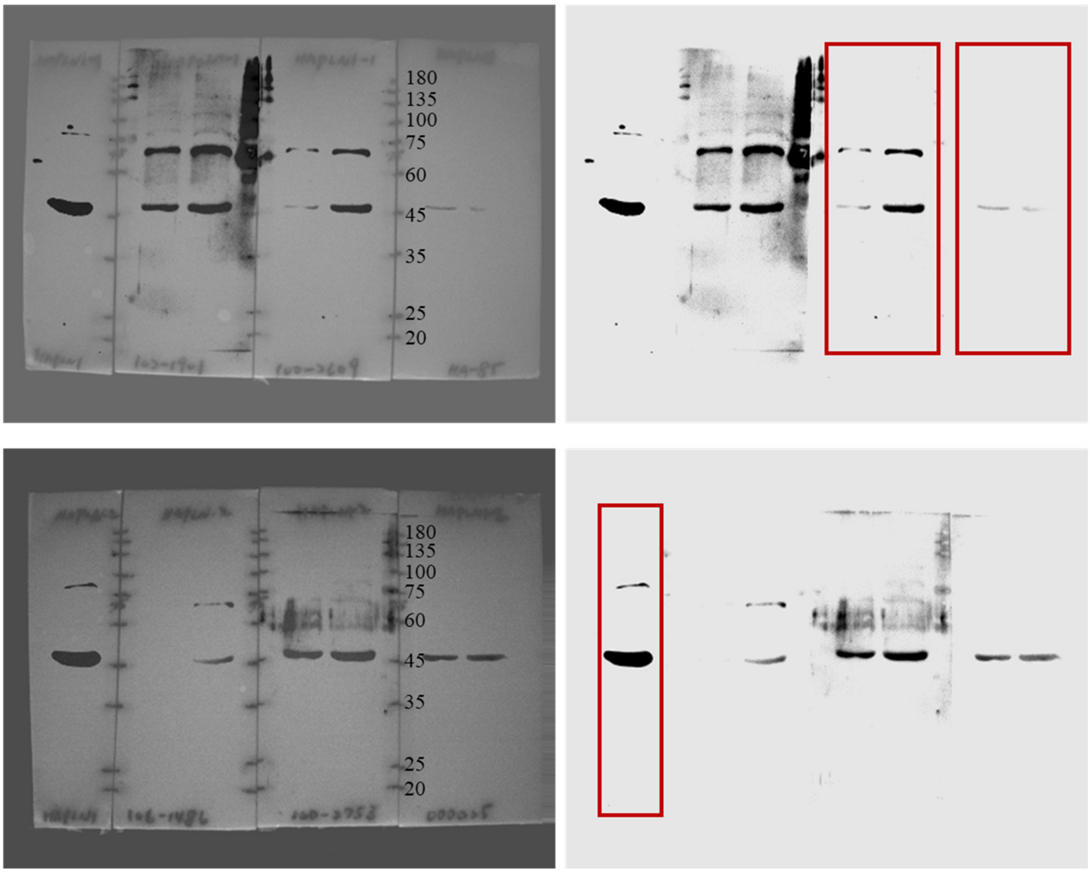

Figure S2. (C) Original images of Figure S1 (C).

(D)

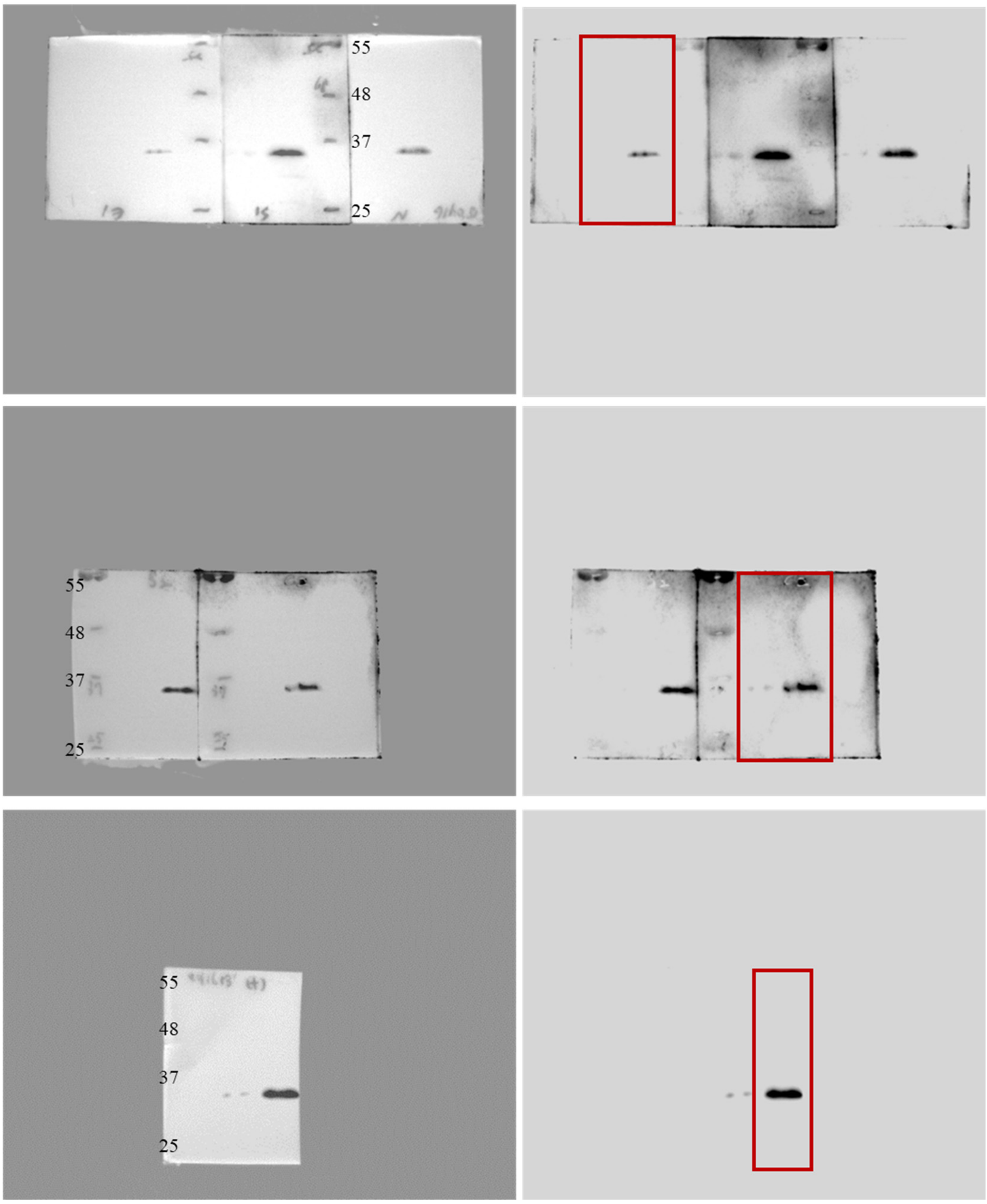

Figure S2. (D) Original images of Figure S1 (D).
