# Supplementary material for: The Implication of Serum Autoantibodies in Prognosis of Canine Mammary Tumors
Source: Animals (Basel). 2022 Sep 18;12(18):2463. doi: 10.3390/ani12182463 (PMC9495273; doi:10.3390/ani12182463)
Supplement: Supplementary file 1 [file animals-12-02463-s001.zip › Table S1.pdf]

Table S1. Clinical characteristics and serum levels of CMT-associated AAbs in 90 CMT dogs enrolled in the overall survival analysis

Characteristics	Case number	TYMS-AAb		IGFBP5-AAb		HAPLN1-AAb		AGR2-AAb	
		Median (range)	Mean ± SD	Median (range)	Mean ± SD	Median (range)	Mean ± SD	Median (range)	Mean ± SD
MT									
BMT	20	0.338 (0.189–0.598)	0.355 ± 0.111	0.294 (0.158–0.502)	0.294 ± 0.085	0.326 (0.203–0.603)	0.350 ± 0.093	0.262 (0.126–0.515)	0.262 ± 0.103
MMT	70	0.383 (0.273–0.733)	0.406 ± 0.099	0.329 (0.179–0.532)	0.338 ± 0.078	0.366 (0.227–1.017)	0.395 ± 0.120	0.275 (0.139–0.533)	0.299 ± 0.111
Clinical stage (MMT)									
I	34	0.379 (0.289–0.733)	0.407 ± 0.107	0.307 (0.179–0.532)	0.329 ± 0.093	0.381 (0.273–1.017)	0.413 ± 0.138	0.274 (0.140–0.533)	0.294 ± 0.106
II	9	0.354 (0.288-0.450)	0.367 ± 0.052	0.323 (0.244–0.442)	0.334 ± 0.053	0.361 (0.227–0.463)	0.355 ± 0.022	0.287 (0.152–0.517)	0.314 ± 0.132
III	16	0.422 (0.273–0.605)	0.413 ± 0.084	0.367 (0.258–0.506)	0.362 ± 0.057	0.329 (0.249–0.545)	0.371 ± 0.093	0.267 (0.147–0.479)	0.293 ± 0.113
IV	7	0.390 (0.282-0.728)	0.439 ± 0.153	0.338 (0.218–0.424)	0.338 ± 0.071	0.474 (0.289–0.661)	0.453 ± 0.133	0.287 (0.139–0.529)	0.315 ± 0.147
V	4	0.398 (0.344–0.462)	0.400 ± 0.052	0.323 (0.230–0.411)	0.323 ± 0.075	0.324 (0.287–0.358)	0.323 ± 0.034	0.279 (0.246–0.395)	0.300 ± 0.070
