# Supplementary material for: The Implication of Serum Autoantibodies in Prognosis of Canine Mammary Tumors
Source: Animals (Basel). 2022 Sep 18;12(18):2463. doi: 10.3390/ani12182463 (PMC9495273; doi:10.3390/ani12182463)
Supplement: Supplementary file 1 [file animals-12-02463-s001.zip › Table S2.pdf]

Table S2. Clinicopathological characteristics of 11 malignant CMT dogs enrolled for assessment of serum AAb levels before and after surgery

Breed	Age (year)	B.W. (kg)	Spayed status[1]	Clinical stage
Chihuahua	12	2.7	Fi	I
Poodle	10	2.7	Fi	I
Dachshund	9	7.1	Fs	I
Maltese	8	2.4	Fs	III
Labrador Retriever	14	32	Fs	IV
Bichon	11	4.8	Fi	I
French Bull dog	7	12.6	Fs	I
Border Collie	8	23.5	Fi	I
Shih-Tsu	10	4.4	Fs	II
Poodle	14	5.4	Fs	I
Dachshund	9	8.5	Fs	I

[1] Fi: intact female; Fs: spayed female
